# Physical strain while wearing personal radiation protection systems in interventional radiology

**DOI:** 10.1371/journal.pone.0271664

**Published:** 2022-07-21

**Authors:** Alexander M. Koenig, Anna Schweer, Daniel Sasse, Robin Etzel, Jonas Apitzsch, Simon Viniol, Rohit P. Thomas, Andreas H. Mahnken

**Affiliations:** 1 Clinic of Diagnostic and Interventional Radiology, Philipps-University of Marburg, Marburg, Germany; 2 Institute of Medical Physics and Radiation Protection, Mittelhessen University of Applied Sciences, Giessen, Germany; 3 Department of Radiology and Nuclear Medicine, Helios Clinic Pforzheim, Pforzheim, Germany; Memorial Sloan Kettering Cancer Center, UNITED STATES

## Abstract

**Objectives:**

Multiple studies show orthopedic health problems for medical staff due to wearing radiation protection aprons. The aim of this study was to evaluate the weight pressure on the shoulder as a marker of physical strain caused by different radiation-protection devices.

**Methods:**

For the weight pressure measurement, a pressure sensor (OMD-30-SE-100N, OptoForce, Budapest, Hungary) placed on the left and right shoulder was used. Wearing different radiation protection systems the force measurement system was used to quantify the weight pressure. Measurements were acquired in still standing position and during various movements.

**Results:**

A mean significant decreasing weight pressure on the shoulder between 74% and 84% (p<0.001) was measured, when the free-hanging radiation protection system was used in comparison to one-piece and two-piece radiation protection aprons and coats. Using two-piece radiation protection aprons, the weight pressure was significantly lower than that of one-piece radiation protection coats. If a belt was used for the one-piece radiation protection coat, the weight pressure on the shoulder was reduced by 32.5% (p = 0.003). For a two-piece radiation protection apron and a one-piece radiation protection coat (with and without belt) a significant different weight pressure distribution between the right and left shoulder could be measured.

**Conclusions:**

The free-hanging radiation protection system showed a significant lower weight pressure in comparison to the other radiation protection devices. Apart from this, use of a two-piece radiation protection apron or addition of a belt to a radiation protection coat proved to be further effective options to reduce weight pressure.

## Introduction

In a large number of investigations, it is necessary for the medical staff to be with the patient during the examination or therapy. Thus, the medical staff is exposed to radiation, predominantly arising from scattered radiation from the patient.

To protect medical staff from this scatter radiation, there are a wide variety of personal radiation protection equipment including different types of personal radiation protection aprons [1].

To ensure efficient radiation protection, personal radiation protection devices are made of materials with a high atomic number. In most cases this includes lead, antimony and/or bismuth, which constitutes to significant weight of the radiation protection aprons ranging from 4 to 11 kg. This weight depends on several factors. The size and the design of the personal radiation protection device are directly related to the weight due to the mass of material used. Also the used material directly influences the weight of the system. For example the combination of antimony and bismuth is lighter than lead alone with comparable radiation protection [2]. Lastly the weight also depends on the used lead equivalence. There is currently no general recommendation for the lead equivalent values of the radiation protection agents to be used, but an AWMF (Association of the Scientific Medical Societies, Germany) guideline is in preparation.

The weight of personal radiation protection aprons leads to increased physical stress of the body. Multiple studies have reported an increase in orthopedic health problems for interventionalists who regularly wear personal radiation protection devices [3–6]. On average, 50% of these studies report orthopedic problems, especially of the spine.

There are reports of significant changes in spinal alignment and of back pain, mostly in young people, who regularly carry a backpack or a school bag [7–10]. These reports indicate that there is a correlation between shoulder strain and back pain.

As there is a wide range of different personal radiation protection devices, the aim of this study is to compare the weight pressure on the shoulders when waering different radiation protection apparels.

## Method and materials

**Fig 1:**
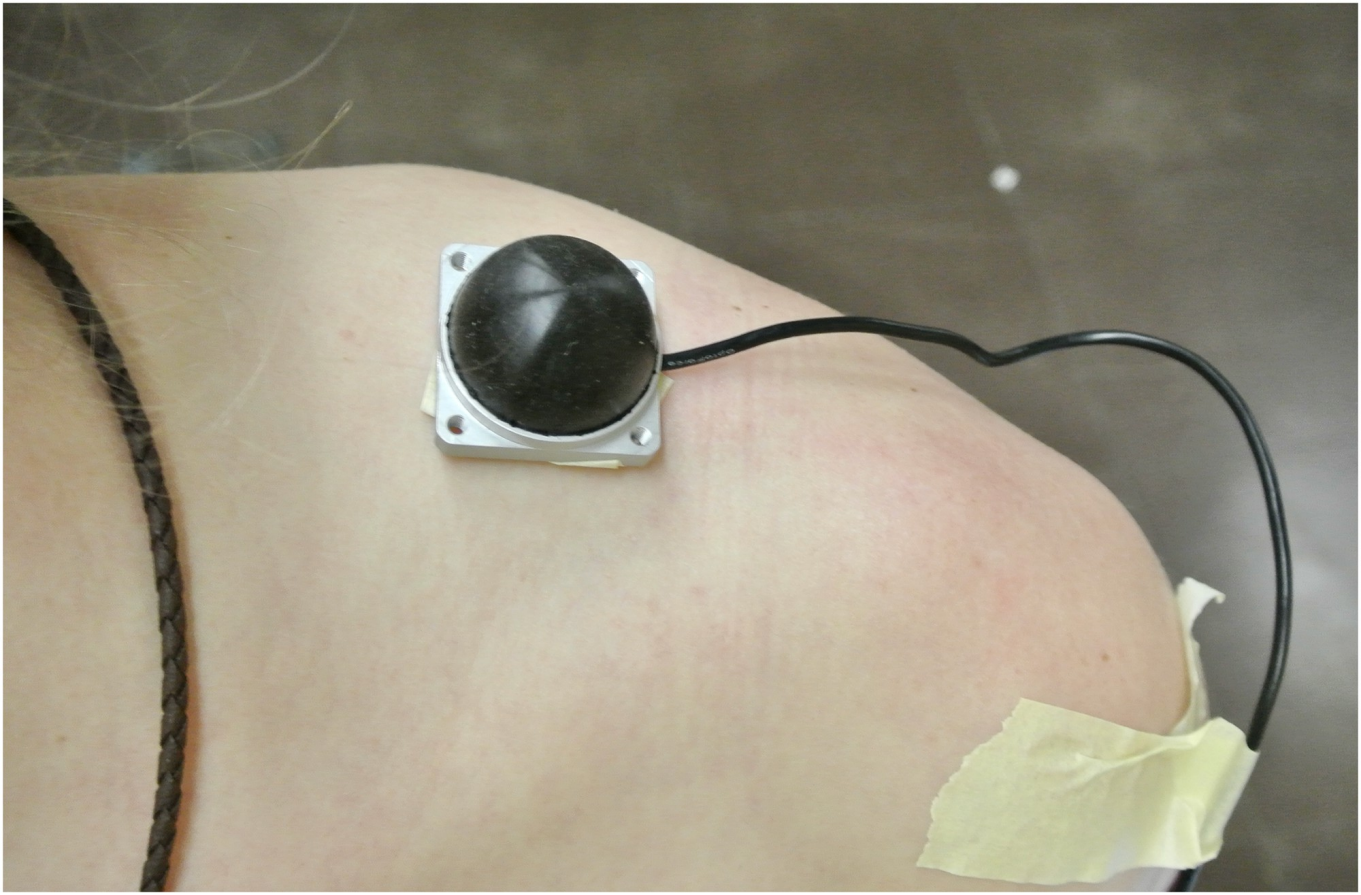
Pressure sensor on the left shoulder.

**Fig 2:**
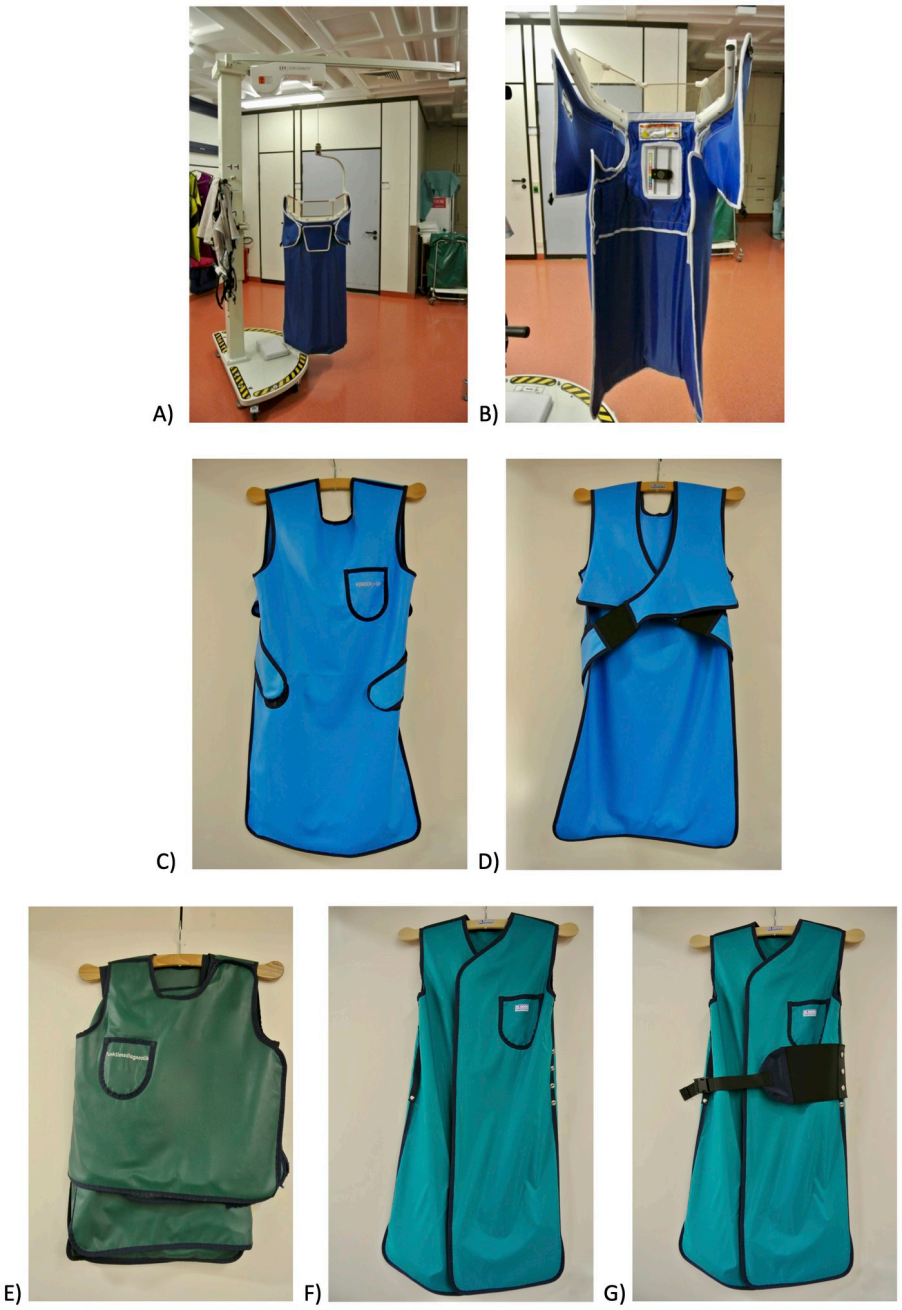
A) and B) system 1: free-hanging radiation protection system (350 kg). C) and D) system 2: one-piece radiation protection apron (4.4 kg). E) system 3: two-piece radiation protection apron (6.6 kg). F) system 4: one-piece radiation protection coat without belt (6.7 kg). G) system 5: one-piece radiation protection coat with belt (7 kg).

A force measurement system was used to quantify the weight pressure on the shoulder while wearing different radiation protection systems and lead aprons. For the weight pressure measurements, a pressure sensor (OMD-30-SE-100N, OptoForce, Budapest, Hungary) with a nominal capacity of 100 N, a full-scale nonlinearity of 2% and a resolution of 6.25 mN was used. The sensor was placed about 3 cm medial to the acromion on the skin above the Trapezoid muscle and was worn on the right as well as the left shoulder (Fig 1). Fig 2 shows the radiation protection systems used, which are described as follows. A free-hanging radiation protection system (350 kg, Zero-Gravity; Biotronik, Berlin, Germany) consisting of a main apron part with a 1.0 mm lead equivalence in the front and 0.5 mm lead equivalence at the side and an acrylic face shield with a 0.5 mm lead equivalence, was analyzed. Here the radiation protection unit is suspended from an arm, which is mounted in the ceiling or on a stand, through which the dead weight can be discharged. Furthermore a one-piece radiation protection apron (front apron 4.4 kg; Dr. Goos Suprema; Heidelberg; Germany) with a lead equivalence of 0.25 mm in the front and side and an open back-side was used. The two-piece radiation protection apron investigated was an apron with skirt and west (6.6 kg, Dr. Goos Suprema; Heidelberg; Germany) with a lead equivalence of 0.5 mm in the front and 0.35 of the back. Another one-piece radiation protection coat without a belt (6.7 kg, Coat apron mantle; Dr. Goos Suprema; Heidelberg, Germany) and a lead equivalence of 0.5 mm in the front and 0.25 mm at the back was also investigated. This radiation protection coat was additionally analyzed with the use of a belt (7 kg).

The weight pressure of the various radiation protection devices on the shoulders was evaluated for the following various postures and movements.

- Standing without any movement.
- Taking a step forward and a step backward.
- Taking a step to the side and back.
- Rotating the upper body right and left by 45°.
- Moving the arms up and down.
- Bending the upper body forward by approx. 30°.

Each movement was practiced by the interventionalists prior to the actual measurements. The personal radiation protection systems were evaluated on 8 persons with an average body weight of 80.5 ± 14.6 kg and a height of 175 ± 10.4 cm.

For data management and statistical evaluation, commercially available analysis programs were used (Microsoft Excel, Version 16.16.18, Microsoft, Redmond, United States and StatPlus:mac LE, V7.1.29, AnalystSoft, Walnut, United States). A variance analysis (ANOVA) was carried out for all radiation protection devices and an explorative two-way post hoc Student t-test was used to estimate the significance between the individual devices. A. p-value of< 0.05 was chosen as the significant standard.

The project was approved by the local Ethical Committee of the Medical Faculty (FB20) of the University of Marburg (file number 11/21). The radiologists who wear the radiation protection aprons in their daily routine and the medical staff were informed about the study and gave their written consent. They were allowed to terminate the study at any time.

## Results and discussion

**Fig 3:**
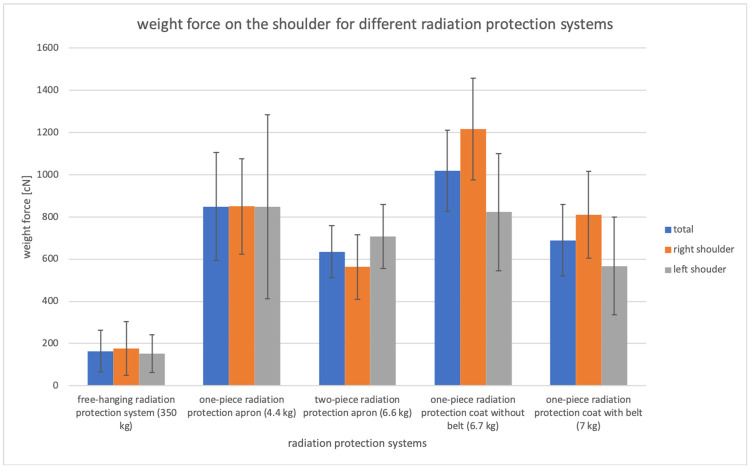
The shoulder load of the different radiation protection aprons averaged and their standard deviation over all movements is shown. The load is listed both as average value over both shoulders and for each individual shoulder. The total weight of the free-hanging radiation protection system is significantly lower than the other systems investigated. Furthermore, the weight of the two-piece radiation protection apron is significantly lower than that of the one-piece radiation protection systems. For the one-piece radiation protection coat with and without a belt a significant difference between the right and left shoulder could be measured.

**Fig 4:**
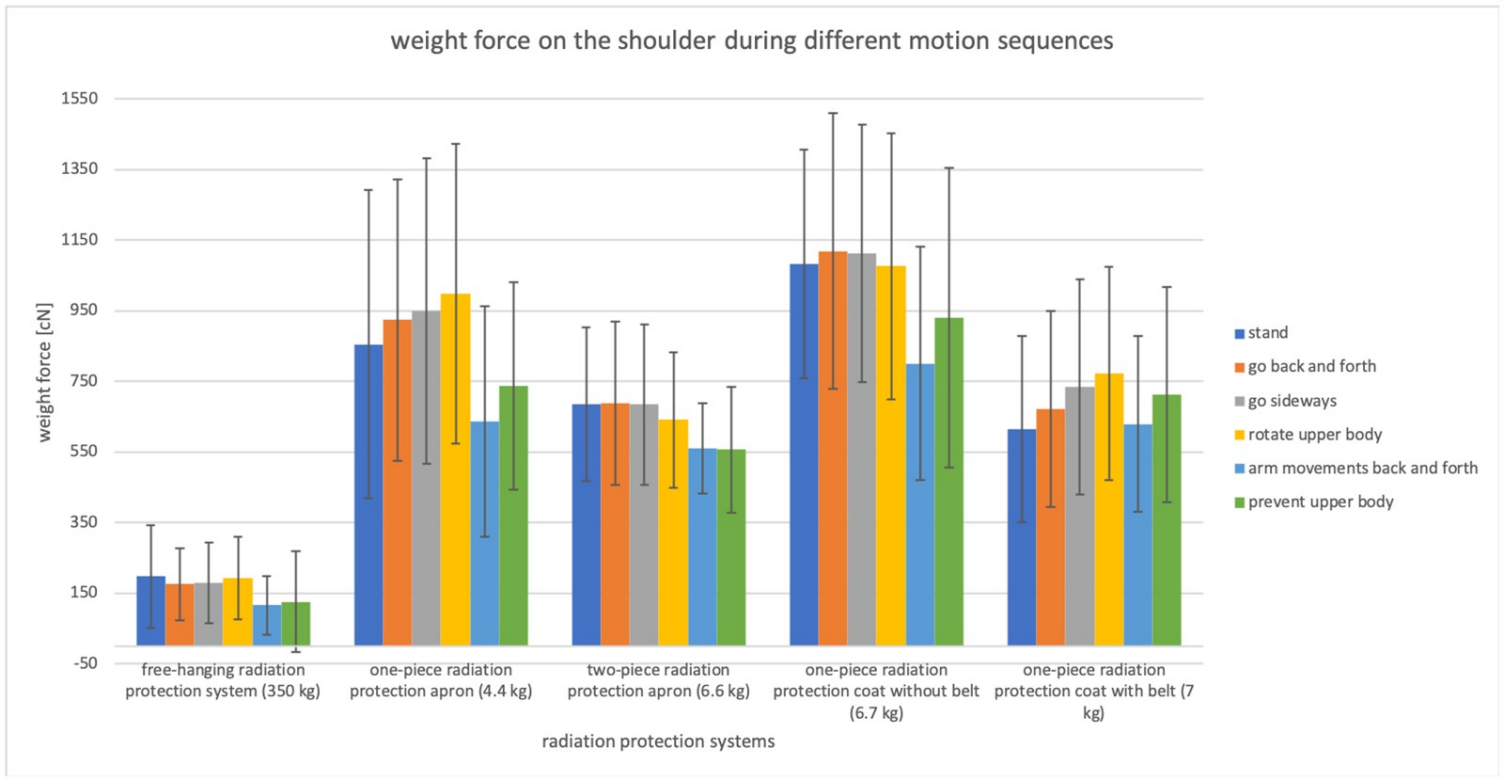
Shoulder loads and their standard deviation during different movements when wearing different radiation protection aprons are shown. The free-hanging radiation protection system showed a significant (p<0.05) difference in the weight on the shoulder while rotating upper body and with arm movement back and forth. For the one-piece radiation protection apron without belt, a significant (p<0.05) weight reduction on the shoulder was measured when the arms were in moving position (moving sequence 5), in contrast to the other moving positions (1–4).

The analysis of variance (ANOVA) showed a significant difference for the weight pressure on both shoulders of the different radiation protection devices (p<0.01). The individual results are summarized in Figs 3 and 4.

The free-hanging radiation protection system showed a significant average lower weight pressure on the shoulder for all movements in comparison to the other radiation protection devices (p<0.01). Compared to the other measured systems a weight pressure reduction between 74% and 84% (p<0.001) was measured. The weight pressure on the shoulder of the two-piece radiation protection apron was 25.2% (p = 0.05) less than that of the one-piece radiation protection apron and 37.7% (p<0.001) less than that of the one-piece radiation protection apron without the use of a belt. There was no significant difference in weight pressure between the two-piece radiation protection apron and the one-piece radiation protection apron with a belt. If a belt was used together with the one-piece radiation protection apron, the weight pressure on the shoulder was reduced by 32.5% (p<0.001) as opposed to the weight pressure without belt.

The free-hanging radiation protection system and the one-piece radiation protection apron showed no significant weight pressure difference between the right and left shoulder. For the two-piece radiation protection apron, a non-significant 25.6% (p = 0.078) weight pressure increase was measured on the left shoulder. For the one-piece radiation protection system without belt a significant 32.3% (p<0.05) weight pressure increase was measured on the right shoulder. If the belt was used, the weight pressure difference was reduced to a significant 29.9% weight pressure (p<0.05).

Only the free-hanging radiation protection system showed a significant (p<0.05) difference in the weight pressure on the shoulder while rotating upper body and with arm movement back and forth. For the one-piece radiation protection apron without belt, a significant (p<0.05) weight pressure reduction on the shoulder was measured when the arms were in moving position (moving sequence 5), in contrast to the other moving positions (1–4). If the belt was used with the one-piece radiation protection apron, the weight pressure on the shoulder decreased so that no significant difference could be measured. A significant reduction of the weight pressure on the shoulder with arm movement (moving position 5) was also measured for the one-piece radiation protection apron (to moving sequence 2–4). The two-piece radiation protection apron showed no significant differences for the movements.

The free-hanging radiation protection system, which compensates almost the entire weight via suspension, put the lowest weight pressure on the shoulders and showed better radiation protection than the others. This is consistent with measurements and experience reports from various publications, which put the main focus on radiation protection [11–13]. But it is also reported that one has to get used to the system and the room concept has to be adapted. Another possibility to reduce weight pressure significantly in comparison to a one-piece radiation protection system is to use a two-piece radiation protection device. Usually a vest and a skirt are used and thereby part of the weight is equally distributed to the hips. This reduction of weight pressure on the shoulder should also reduce the strain to the spine. However, these measures do not reduce the weight on the lower extremities. It can therefore be assumed that not all orthopedic problems like of the hip, knee and ankle that were reported by Klein et. al. will change through the use of this system [4]. If a one-piece radiation protection system is used, the weight pressure on the shoulder can be significantly reduced through the use of a belt. But here also, the weight is shifted only to the hip and no changes of strain in the lower extremities are to be expected. It could be shown that, depending on the radiation protection device used, the weight pressure can sometimes be much higher, and it can be assumed that this also increases the physical strain. Such loads are intensively investigated in the literature with carrying backpacks. Carrying such a backpack with 17% of the body weight, Bettany-Saltikov et. al. report significantly decreased thoracic kyphosis in the sagittal plane [10]. This can lead to back pain in the long run and is also discussed extensively in the literature [9, 10, 14].

The two-piece radiation protection apron and the one-piece radiation protection apron with and without belt showed a significant difference of weight pressure on the right and left shoulder. The reason for this could be the apron´s overlap in the front. The two-piece radiation protection apron overlaps on the left, where a higher weight pressure was measured. The one-piece radiation protection apron overlaps on the right where a higher weight pressure was measured. Furthermore, the different weight pressure could only be reduced with the additional use of a belt. In the case of unilateral shoulder strain, Bettany-Saltikov et al. reports significant increase in thoracic lateral curvature in the frontal plane and decreased thoracic kyphosis in the sagittal plane [10]. Furthermore they showed one-side lead deviations in all planes which may cause adverse stress and strain on spinal structures and ultimately lead to pain [14]. This indicates that one-sided shoulder`s strain should be avoided in order to prevent orthopedic problems, especially of the spine.

Some radiation protection devices showed a significant weight pressure reduction on the shoulder when the arms were in moving position. This is most likely due to the fact that the radiation protection system was lifted off the sensor. The non-significant reduction in weight pressure when bending forward results from the fact that the lead apron rests more on the back, thus reducing the weight pressure on the shoulder. Further investigations, such as simulations, are required to validate these facts.

One of the study`s limitations is that the radiation protection devices were not adapted to the test persons. But this reflects the daily routine in many clinics, where only certain sizes are available and these then have to be used. Using the right size not only improves the radiation protection, but also the physical strain could be reduced. Another limitation is that the pressure sensor is only a punctual measurement and does not measure the weight pressure on the whole shoulder. However, this measured value should correlate and should be comparable for all the radiation protection devices.

In summary, it could be shown that the weight pressure lead on the shoulder is significantly different between different radiation protection systems. There is also a weight pressure difference between the right and left shoulder in some systems. Thus, an exact selection regarding size, fit and field of application of the radiation protection device used should be made to avoid orthopedic problems.

## Conclusion

The free-hanging radiation protection system showed a significant lower weight pressure in comparison to the other radiation protection devices. Apart from this, use of a two-piece radiation protection apron or addition of a belt to a radiation protection coat proved to be further effective options to reduce weight pressure. This reduction in weight pressure on the shoulder should also lead to a reduction in physical stress, especially on the spine. Some radiation protection systems showed different weight pressure of the right and left shoulder, which could additionally lead to orthopedic problems.

## Supporting information

S1 Data (XLSX)
